# Characterisation of Uruguayan Honeys by Multi-Elemental Analyses as a Basis to Assess Their Geographical Origin

**DOI:** 10.3390/foods8010024

**Published:** 2019-01-11

**Authors:** Verónica Berriel, Patricia Barreto, Carlos Perdomo

**Affiliations:** 1 Centre for Applications of Nuclear Technology in Sustainable Agriculture, Soil and Water Department, Agronomy College, University of the Republic. Av. Garzón 809, CP 12.900 Montevideo, Uruguay; 2 Soil Fertility Lab, Soil and Water Department, Agronomy College, University of the Republic. Av. Garzón 780, CP 12.900 Montevideo, Uruguay; pbarreto@fagro.edu.uy; 3 Soil and Water Department, Agronomy College, University of the Republic. Av. Garzón 780, CP 12.900 Montevideo, Uruguay; chperdom@fagro.edu.uy

**Keywords:** honey, mineral content, discriminant analysis

## Abstract

In this work, we evaluated the possibility of predicting the geographic origin of Uruguayan honeys using discriminant analysis (DA) on mineral concentration. Although the DA results appeared to discriminate between honeys from the south, central and north, the subsequent cross-validation analysis did not confirm this result. We also compared honeys from Uruguay and the Buenos Aires province (Argentina) using DA on mineral composition data. In this case, a clear difference between these two origins was observed. It seems possible to differentiate between Uruguayan honeys and those produced in a neighbouring country based on multivariate statistical methods.

## 1. Introduction

Honey is the third most adulterated food in the world; this problem currently includes the deliberated mislabelling of a honey’s origin [1,2]. This counterfeiting is triggered by the strong price variation of honey according to its origin [1], which has occurred for some time at the international level [3]. For this reason, honey trading is highly regulated, and these regulations are imposed by both importer and exporter countries. The legislations of Uruguay [4] and Southern Common Market (MERCOSUR) [3] include the obligation to state the floral and/or geographical origin of each honey consignment, but this declaration is difficult to verify and can be easily distorted. Thus, there is a need to strengthen traceability declarations with confirmatory analytical information.

In recent years, a large number of models have been used and developed in order to assign or predict the geographical or botanical origin of honeys; to this end, different chemical variables have been used in conjunction with multivariate statistical methods [5,6,7]. Among the many chemical variables used for this purpose are those related to the concentration of minerals in honeys [8,9,10,11]. Multivariate statistical methods have been used to reduce all of these variables to a small number of vectors composed of uncorrelated variables, allowing the identification of honey groups of similar composition as well as the splitting or assigning of new samples to any of these groups [12]. Among the most commonly used multivariate statistical methods in honey-related studies are principal component analysis (PCA), discriminant analysis (DA), cluster and nearest neighbour analyses, partial least squares regression, neural networks and multiple logistic regression (MLR) [1].

These statistical techniques have been extensively used to classify honey according to its geographical origin, for which the studies conducted in Italy [10] and Turkey [13] can serve as examples; in these studies, the researchers achieved the correct classification of honey samples originating from different areas by applying PCA to the mineral concentration values of the samples. Similarly, Bontempo et al. [14] reported that it is possible to differentiate the production area (north, central or south) of chestnut honey by performing PCA on the data regarding the mineral concentration of honey and the stable isotopic signature of its protein (C, N and H). At the regional scale, Baroni et al. [15] applied DA to the concentration values of Rb, B, U, Na, La and Zn as well as to the ratios of K/Rb and ^87^Sr/^86^Sr of honey samples produced in different regions of Argentina (the provinces of Buenos Aires, Córdoba and Entre Ríos). 

Local research on this topic is relevant because if this identification were feasible, it would increase importers’ confidence in such products. Therefore, the purpose of this study was to evaluate the possibility of identifying the geographical origin of Uruguayan honeys based on their mineral compositions. 

## 2. Materials and Methods

### 2.1. Sample Collection

Honey samples were collected from 25 apiaries located in different regions of Uruguay (30° and 35° S, 53° and 58° W). With regard to the apiary selection, we selected three climate regions of the country—south (S), central (C) and north (N)—according to the average annual rainfall. The area distribution of precipitation was between 1100 and 1200 mm in the south, 1200 and 1300 mm in the centre and 1300 and 1400 mm in the north region. 

In 2008, from each apiary, a composite sample of honey was taken from all the hives that existed. In all the areas, honey with the same botanical origins was sampled (eucalyptus, prairie and native woody vegetation). The samples were transported to the laboratory in airtight containers and stored in conditions of darkness and controlled temperature (4 °C). Immediately before the analyses, the samples were allowed to equilibrate to room temperature (25 °C) and were homogenised.

### 2.2. Sample Preparation and Analysis

A 5 g honey sample was placed in a previously calcined crucible to determine the concentrations of K, Na, Ca, Mg, Fe, Mn, Zn, and Cu. To avoid foam leakage, the crucible with the sample was progressively heated on a hot plate until the content became blackened. Afterwards, the sample was calcined in a muffle furnace (Thermolyne Thermo Scientific FA1730, Dubuque, IA, USA) at 550 °C until a constant weight was reached. The resulting ash was dissolved in a 10 mL mixture (1:1) of 1 M HCl and 1 M HNO_3_ and brought to a final volume of 100 mL with distilled water. The determination of the elements was performed directly in the resulting solution. In the case of K and Na, determinations were made by flame photometry, whereas the other elements were determined by atomic absorption spectroscopy; a Perkin Elmer 373 atomic absorption spectrometer (Perkin Elmer Corp., Analytical Instruments, Norwalk, CT, USA) was used in both cases. The analysis was repeated three times for each sample.

### 2.3. Statistical Analysis

Univariate analyses (ANOVA and the Tukey test) were performed for each analytical variable using the zone as a classifier variable. Backward stepwise linear discriminant analysis was used to find the combination of independent variables (mineral concentration) that maximised the variance between the pre-established geographical groups and at the same time minimised the intragroup variance [16]. The proportion of samples assigned to each of the original groups was one of the criteria used to evaluate the robustness of the DA model. This criterion was also used to evaluate the cross-validation result; this stage consisted of a series of DA reruns (equal to the number of observations), and in each of these reruns, one of the samples was omitted. The closer this proportion was to 1, the more the model was considered robust. All statistical analyses were performed using the XLStat program (Addinsoft SARL©, 2018, Paris, France).

## 3. Results and Discussion

### 3.1. Mineral Concentration

The ranking of the mean mineral concentration in Uruguayan honey was K > Ca > Na > Mg > Fe > Mn > Zn > Cu (Table 1). According to ANOVA, there were no significant differences in mineral concentration between honeys originating from the different geographical areas defined in this work, probably because different soil types coexisted within each zone. These results are not unexpected, as the criterion used for zone delimitation was purely geographic. Thus, it is possible that part of the variation in mineral concentration observed within each zone could be related to the variation in soil type, since it is well known that the geochemical and geological characteristics of soil affect the mineral composition of both flora and honey [17,18]. 

Uruguayan honeys had higher concentrations of Ca but less Na and Mg than Argentinean multiflora honeys from the provinces of Buenos Aires, Entre Ríos and Córdoba as reported by Conti et al. [19]. The same result was observed when our samples were compared with those of another survey conducted in the province of Córdoba by Baroni et al. [18], although these authors later observed that Ca was more concentrated than K in another set of honey samples collected from the Buenos Aires province [15]. In contrast, Pisani et al. [20] from Italy and Habib et al. [21] from the United Arab Emirates reported a similar ranking of the first four minerals to that observed for the Uruguayan samples.

The K concentration varied from 30 to 930 mg/kg (382 ± 242), with a range that was narrower than that reported for honey from Morocco [22], the Canary Islands [8], Argentina [19], and the United Arab Emirates [21]. The maximum K values observed in these countries ranged from 2388 to 7030 mg/kg. The range of Ca was between 40 and 120 mg/kg (71 ± 26), which was slightly lower than that that for honeys from Spain [8], and Portugal [23], but higher than that of honey from the United Arab Emirates [21], which ranged from 7 to 248 mg/kg. In contrast, the Ca range observed in Argentinean honeys was narrower than ours, varying between 2 and 19 mg/kg [24]. 

The observed variation in Na concentration in our Uruguayan honeys was between 20 and 100 mg/kg (56 ± 21). Honeys from Argentina showed a similar range (5 to 106 mg/kg) [19], but those from the United Arab Emirates, Spain, and Portugal were more variable and had much higher maximum values. In the first case, this variation was from 9 to 258 mg/kg [8] and in the second set it was from 90 to 728 mg/kg [22]. In the United Arab Emirates, this range was from 7 to 532 mg/kg [21].

The concentration of Fe varied from 1 to 13 mg/kg (3 ± 2). This concentration was similar to United Arab Emirates honeys, which varied from 1 to 111 mg/kg [21], while it was 4 mg/kg for Argentinean honeys [19]. 

In the case of Mg, the concentration was between 1 and 10 mg/kg (3 ± 2); this concentration occurred in a narrower range than that reported for Moroccan, Spanish, and United Arab Emirates honeys, whose ranges were from 5 to 220 [22], 7 to 165 [8], and 2 to 161 mg/kg [21], respectively. The range in variation of Mn was 0.2 to 4.2 mg/kg (1.5 ± 1.0); this range was greater than that reported for honeys from Morocco [22], Argentina [19], and the United Arab Emirates [21], where the maximum values were 11.5, 10.3, and 8.8 mg/kg, respectively.

In the case of Zn, the concentration ranged from 0.2 to 7.0 mg/kg (3.1 ± 1.3), which was similar to that reported by Habib et al. [21] and for the United Arab Emirates (0.3 to 6.7 mg/kg). However, the maximum values found in our study were lower than those reported by Terrab et al. [22], Hernández et al. [8], and Tuzen et al. [9] for honeys from Morocco, Spain, and Turkey, respectively, with values of 12.5, 19.1, and 12.7 mg/kg, respectively. At the other extreme, the concentration range reported for Argentinean honeys tended to be lower than ours, reaching a maximum of 2.7 mg/kg [19]. 

The Cu concentration in our samples ranged from 0.2 to 5 mg/kg (2.0 ± 1.2). This range was similar to that observed in Argentina [19], Turkey [9], Morocco [22], and Slovenia [25], with ranges from 0.1 to 1.2, 0.25 to 2.41, 0.1 to 4.7, and 1.4 to 2.7 mg/kg, respectively. However, the range in our samples was lower than the range of 10.0 to 29.5 mg/kg reported for Italian honeys by several authors [20,26,27,28]. In contrast, our samples had higher Cu concentrations compared with those of Irish honeys, with a reported maximum value of 0.2 mg/kg [29], and with those of the Canary Islands honeys, where the reported range was from 0.1 to 1.7 mg/kg [8]. Therefore, despite some minor differences, these results indicate that the ranges of mineral concentration of the Uruguayan honey reported in this work are within the ranges of internationally reported values.

### 3.2. Discrimination of Honey Origin

DA was used to identify the equations that could best differentiate the honeys obtained from different Uruguayan regions. According to the stepwise discriminant procedure, the Lambda Wilks test was significant (*p* = 0.004), indicating that the DA model was acceptable and thereby allowing the classification of samples originating from the south (S), centre (C) and north (N) of Uruguay. Despite these auspicious results, and because the first and second canonical functions explained 72.6% and 26.4% of the total variability, respectively, the prediction ellipses of the three zones clearly overlapped and did not facilitate a clear separation between honeys from each geographical origin. A posterior cross-validation analysis confirmed that the resolution power of this model was low, as error rates of 25%, 50% and 29% were obtained for the samples originating from the S, C and N, respectively (Table 2). 

Another DA was performed based on the previous results, but in this case, samples from the S and C were classified into a single group (S and C). The resultant model was statistically significant (p of Lambda Wilks test = 0.0004) and the first discriminant function explained 100% of the variability, allowing the classification of honeys from N, C and S with 15% global error rates (Table 2). However, as in the previous DA, the error rates of the subsequent cross-validation were higher, 29% and 25% for the N group and S & C group, respectively. In turn, the global cross-validation error rate was 23%. These high error rates indicated that the resulting DA model was not robust enough as to be used to identify honey produced in different macro-regions of Uruguay. In contrast, a DA model developed in Spain, also based on honey´s mineral content, allowed the authors to distinguish honey from the Canary Islands from honey collected in other Spanish regions with an error rate of only 5% [9]. The local Uruguayan results were likely due in part to the relatively small geographical extension of Uruguay, and to soils being highly variable in each area. Despite this result, it is important to stress that also in another study done locally has it been possible to discriminate between honeys from different floral origins [12,30]. Recently, using quadratic DA, it has been possible to discriminate between different types of honey in Uruguay as polyfloral and eucalyptus through isotopic and physicochemical variables, achieving 100% correct allocation both at the training stage and the cross-validation stage [30].

### 3.3. Differentiation of Uruguayan Honey from Argentinian Honey from Buenos Aires Province

**Figure 1:**
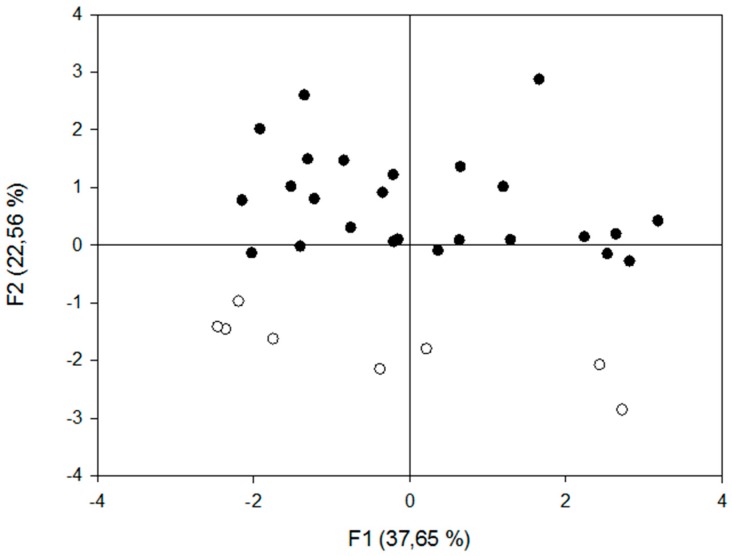
Distribution of honey samples from Uruguay (in black) and Buenos Aires (in white) based on the first (F1) and second (F2) functions of the principal component analysis.

The honey samples from Uruguay collected in this study were compared with samples from the Buenos Aires province. This Argentinean province was a relevant benchmark, since it is located nearby and has a geographical size and climate similar to Uruguay. The simplest and most robust DA model (*p* < 0.05) included the significant variables Zn and Cu. Only one discriminant function was needed to explain 100% of the observed variation. Centroids were −1.62 and 1.62 for Buenos Aires and Uruguay, respectively. The resulting algorithm (Equation (1)) discriminated Uruguayan from Buenos Aires honeys with a low cross-validation error rate (6%). Through the graphical representation of the analysis of principal components, it was possible to visualise the grouping of honeys (Figure 1) according to their geographical origin (Uruguay vs Buenos Aires). Thus, the different mineral compositions of honeys from these two nearby regions were enough to successfully discriminate between them.

(1) f(x)=0.784[Zn]+0.788[Cu]−2.614

## 4. Conclusions

In conclusion, in this work, it was not possible to discriminate between honey produced in macro-regions inside Uruguay based on only their mineral compositions. However, it seems feasible to discriminate between Uruguayan honeys as a single group from those produced in the Buenos Aires province. In this case, the model obtained achieved 94% correct allocation both at the training stage and the cross-validation stage. It is necessary to stress, however, that these results must be evaluated with caution due to the low number of samples that were analysed. In the future, it will be interesting to apply this technique to discriminate between honeys produced in the different micro-regions of Uruguay.

## Figures and Tables

**Table 1:** Mineral concentration (mean ± SD ^1^) of several sets of honey samples grouped by geographical origin.

Source of Variability	n ^2^	Mineral Concentration							
		K	Ca	Na	Mg	Fe	Mn	Zn	Cu
		mg/kg							
**Region**									
**North**	9	347.48 ± 178.99	64.45 ± 25.50	49.18 ± 17.32	2.68 ± 2.96	2.40 ± 1.70	1.43 ± 0.93	2.64 ± 0.87	2.33 ± 0.71
**Central**	11	449.89 ± 293.31	77.07 ± 27.34	65.63 ± 21.15	2.97 ± 1.92	4.19 ± 3.18	1.27 ± 0.73	3.06 ± 1.76	1.41 ± 1.21
**South**	5	440.95 ± 203.79	77.60 ± 28.81	52.96 ± 23.87	2.83 ± 2.05	3.16 ± 1.87	2.21 ± 1.51	3.30 ± 1.14	2.57 ± 1.52
**Buenos Aires**	8	458.06 ± 364.17	ND ^3^	39.32 ± 36.82	13.33 ± 9.76	3.01 ± 0.81	ND ^3^	1.06 ± 0.51	0.20 ± 0.06

SD ^1^ = standard deviation; n ^2^ = number of samples; ND ^3^ = not determined.

**Table 2:** Classification of Uruguayan honey samples with different geographical origins and the percentage of correctly classified observations made using discriminant analysis.

	Predicted Group Membership (%)						
	Geographical Origin						
	First Run Analysis				Second Run Analysis		
**Method**	N ^1^	C ^2^	S ^3^	Overall	N	C & S	Overall
**Original**	85	87	100	91	86	83	85
**Cross-validation**	71	50	75	65	71	75	73

Geographical origin: N ^1^ = North, C ^2^ = Central, S ^3^ = South.
